# Iron, oxygen, and the pulmonary circulation

**DOI:** 10.1152/japplphysiol.00179.2015

**Published:** 2015-06-11

**Authors:** Matthew C. Frise, Peter A. Robbins

**Affiliations:** Department of Physiology, Anatomy and Genetics, University of Oxford, Oxford, United Kingdom

**Keywords:** iron, hypoxia, hypoxic pulmonary vasoconstriction, hypoxia-inducible factor, high-altitude illness

## Abstract

The human pulmonary vasculature vasoconstricts in response to a reduction in alveolar oxygen tension, a phenomenon termed hypoxic pulmonary vasoconstriction (HPV). This review describes the time course of this behavior, which occurs in distinct phases, and then explores the importance for HPV of the hypoxia-inducible factor (HIF) pathway. Next, the HIF-hydroxylase enzymes that act as molecular oxygen sensors within the HIF pathway are discussed. These enzymes are particularly sensitive to intracellular iron availability, which confers iron-sensing properties on the HIF pathway. Human studies of iron chelation and supplementation are then reviewed. These demonstrate that the iron sensitivity of the HIF pathway evident from in vitro experiments is relevant to human pulmonary vascular physiology. Next, the importance of iron status in high-altitude illness and chronic cardiopulmonary disease is explored, and the therapeutic potential of intravenous iron discussed. The review concludes by highlighting some further complexities that arise from interactions between the HIF pathway and other intracellular iron-sensing mechanisms.

“*Since life's addiction to iron transcends the oxygenation of the Earth's atmosphere, living things must be protected from the potentially dangerous mix of iron and oxygen. The human being possesses grams of this potentially toxic transition metal, which is shuttling through his oxygen-rich humor”* (77).

this mini-review is concerned with the way in which iron and oxygen interact in the setting of the pulmonary circulation. Even a brief glance at the literature reveals frequent references to the reactivity of iron and oxygen and how dangerous they can be in combination. Nevertheless, many key cellular and physiological processes rely on their successful interaction. Most obviously, the oxygen-transport properties of hemoglobin depend on the binding of oxygen to ferrous iron within heme. Within the mitochondrion, iron is an essential component of several subunits of the electron transport chain. Furthermore, many members of a large family of enzymes, the 2-oxoglutarate-dependent dioxygenases—recognized, inter alia, as central to cellular hypoxia sensing—also use ferrous iron as a cofactor.

The erythropoietic response to hypobaric hypoxia helpfully demonstrates one link between iron and oxygen in physiology. Over a century ago, the striking relationship between hematocrit and altitude of residence was demonstrated by Mabel FitzGerald, who made careful measurements in individuals residing over a range of elevations as part of the Pike's Peak expedition (25). What underlies this relationship? Alveolar hypoxia as a consequence of the fall in atmospheric pressure with ascent leads to arterial hypoxemia. Interstitial fibroblasts of the kidney respond to the fall in oxygen content of the blood and secrete erythropoietin, stimulating erythrocytosis, which requires the supply of large amounts of iron for new red blood cells. The resulting increase in hematocrit restores tissue oxygen delivery to a degree, but brings with it a need for more iron. Remarkably, it is not a fall in circulating iron levels that is the initial signal for the body to absorb more iron from the gut and liberate iron stores (90); it is hypoxemia itself via an as yet incompletely characterized, bone marrow-dependent pathway, which suppresses the iron-regulatory hormone hepcidin (31, 45, 50). In this setting, hypoxia signifies to the body that there is an impending iron requirement. A low-oxygen signal is translated as a low-iron signal. Given that expansion of the circulating red blood cell mass is a huge sink for iron, the advantages of this arrangement are clear. However, as shall be discussed, it also turns out to be the case that the converse is true: iron deficiency generates a hypoxia signal, with wide-ranging implications for human physiology, particularly high-altitude physiology, and many human diseases.

## MEASURING THE RESPONSE OF THE PULMONARY CIRCULATION TO HYPOXIA

Nearly a century ago, Haldane recognized that the lungs ought to employ a mechanism to match delivery of deoxygenated blood to well-ventilated lung regions (35). Two decades later, the phenomenon of hypoxic pulmonary vasoconstriction (HPV) was demonstrated in anesthetized cats (96) and subsequently in humans (57, 101). While the role of HPV in determining regional pulmonary blood flow at rest in healthy humans continues to be the subject of investigation (7, 8, 22), what is certain is that the pulmonary vasculature is highly responsive to hypoxia. This is well demonstrated by pathological situations in which there is regional (42) or global (100) alveolar hypoxia.

### 

#### Quantifying HPV.

Which variable ought to be measured when attempting to quantify HPV—is it more appropriate to measure pulmonary arterial systolic pressure (PASP) or pulmonary vascular resistance (PVR)? Neither of these variables is a direct measure of pulmonary vascular smooth muscle tone, which is of most interest physiologically. The change in cardiac output (CO) that occurs as a result of hypoxia may affect both PASP and PVR directly, and this is an added complication. The pulmonary circulation is remarkable in its ability to accommodate a severalfold increase in CO during exercise without any great change in the arteriovenous pressure difference across the lung. Thus PVR varies greatly as a function of CO, and so does not make a good index of changes in pulmonary vascular smooth muscle activity. In contrast, changes in PASP are themselves much less affected by variations in cardiac output, and therefore provide a better measure of changes in pulmonary vascular tone. This topic is considered in more detail elsewhere (10, 11, 20).

The gold-standard technique for investigating pulmonary hemodynamics is pulmonary artery catheterization. Although this undoubtedly plays a key role in the clinical assessment of patients with pulmonary hypertension and right heart disease, and is appropriately used in such individuals in the setting of clinical trials, the associated complications make it unattractive as a technique for use in healthy volunteer physiology studies. Much of the more recent work examining the pulmonary circulation during hypoxia has therefore employed transthoracic echocardiography, which is noninvasive and gives measurements that correlate very closely with those obtained from right heart catheterization (3, 21, 62, 86, 104). One widely-used technique involves measuring the maximum velocity of a small regurgitant jet of blood through the tricuspid valve, which occurs during systole in the majority of individuals. Using Bernoulli's equation, this velocity can be used to estimate the pressure difference (ΔPmax) between the right ventricle and right atrium. Since right atrial pressure (RAP) is not significantly altered by hypoxia (32), ΔPmax itself offers a convenient index of HPV, although an estimated RAP may be added to give PASP (17, 48).

#### Changes in HPV over time.

**Fig. 1.**
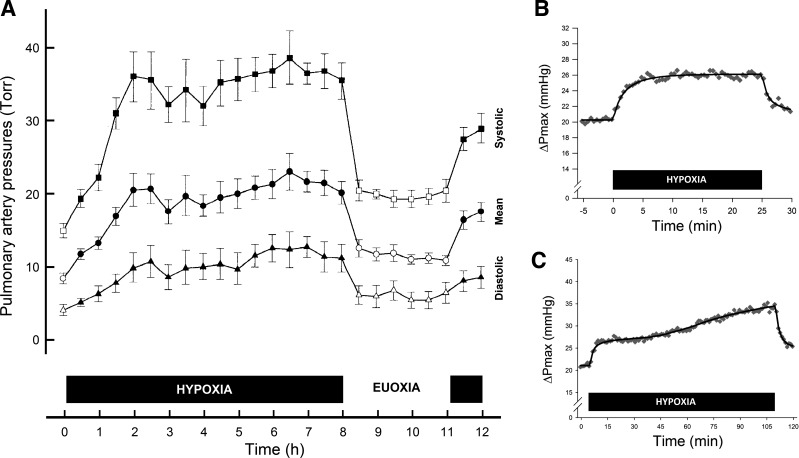
*A*: the rise in pulmonary artery pressure (PAP) during 8-h sustained alveolar hypoxia [end-tidal Po_2_ (Pet_O_2__) = 50 Torr] measured using pulmonary artery catheterization in 6 healthy male volunteers. Squares, pulmonary arterial systolic pressure (PASP); circles, mean PAP; triangles, diastolic PAP. Open symbols indicate measurements in euoxia, filled symbols measurements in hypoxia. On return to euoxia at 8 h, PASP initially remains elevated; if a further short hypoxic challenge is given, the rise in PASP is considerably more brisk than that observed at the beginning of the experiment. *B*: rise in ΔPmax during, and rapid return towards resting levels after, 25-min alveolar hypoxia (Pet_O_2__ = 50 Torr) in a group of 12 healthy individuals (6 male, 6 female). *C*: second phase of HPV seen to occur around 40-min into a period of 105-min alveolar hypoxia (Pet_O_2__ = 50 Torr) in a group of 7 healthy individuals (5 male, 2 female). It can be seen that, in contrast to the brief hypoxia exposure in *B*, ΔPmax remains elevated after cessation of hypoxia. Measurements in *B* and *C* made using transthoracic Doppler echocardiography. [Modified from Dorrington et al. (23) and Talbot et al. (88) with permission.]

The effects of changes in alveolar gas tensions on the pulmonary vasculature may be conveniently studied by using either an end-tidal forcing system (70), where the participant breathes through a mouthpiece, or for longer studies a normobaric chamber (38). Figure 1*A* shows the time course of the rise in PASP in response to a sustained period of alveolar hypoxia in a normobaric chamber, characterized using pulmonary artery catheterization (23). It is apparent that the pulmonary vasculature has been changed in some way as a consequence of this sustained hypoxic stimulus, since if a further hypoxic challenge is delivered while euoxic PASP remains elevated, the resulting acute response is more marked: acclimatization has occurred.

Further temporal resolution of the early stages of HPV has been provided using an end-tidal forcing system and transthoracic echocardiography (88). With the onset of alveolar hypoxia, a first phase of HPV is rapidly detectable and becomes maximal within a few minutes (Fig. 1*B*); following this initial brisk response, a second phase can be demonstrated after ∼40 min (Fig. 1*C*). If the pulmonary vasculature is exposed to a brief hypoxic stimulus, less than or equal to the duration of the first phase of HPV (Fig. 1*B*), then on return to euoxia PASP quickly returns to preexposure levels. If the hypoxic stimulus continues such that the second phase of HPV is manifest (Fig. 1, *A* and *C*), then it takes considerably longer for PASP to fall back to baseline levels (10, 23, 81, 88). The magnitude of this effect depends on the duration of hypoxia; when this extends beyond hours into days and weeks, pulmonary vascular remodeling occurs, such that inhalation of supplemental oxygen has little effect (32).

## MECHANISMS UNDERLYING HPV: INSIGHTS FROM GENETIC ALTERATIONS OF OXYGEN SENSING

The underlying processes responsible for the mediation and modulation of HPV are the subject of intense research, and the literature on the subject is vast (58, 87). At the most fundamental level, HPV involves inhibition of oxygen-sensitive potassium channels, depolarization of pulmonary artery smooth muscle cells, and calcium influx through voltage-gated calcium channels causing vasoconstriction. Setting aside this immediate response of the pulmonary vasculature to alveolar hypoxia, the observation that the second phase of HPV brings with it a temporary change in the behavior of the pulmonary circulation suggests that alterations in gene expression may be important during this component of the pulmonary vascular response to hypoxia. An obvious candidate for orchestrating at least some of this is the hypoxia-inducible factor (HIF) pathway (78).

### 

#### The HIF pathway.

HIF acts as a transcription factor at hypoxia-response element (HRE) sites in the genome when bound as a dimer consisting of one HIFα and one HIFβ subunit (98). HREs regulate the expression of hundreds of genes including those involved in erythropoiesis, angiogenesis, and metabolism (44, 76). The HIF pathway provides cells with the machinery needed to sense and respond to changes in oxygen tension, since the HIFα protein is a target for hydroxylation at several amino acid residues by enzymes with an absolute requirement for dioxygen—prolyl-hydroxylase domain enzymes (PHDs) and an asparaginyl hydroxylase. Depending on the site of hydroxylation, HIFα is either tagged for polyubiquitination and proteasomal degradation, or, if it escapes this process, inhibited from recruiting coactivators required for promoter activity at HREs (44). Since these inhibitory effects rely on hydroxylation which in turn depends on the abundance of oxygen, hypoxia quickly leads to an increase in the intracellular level of HIFα, dimerization with HIFβ, and recruitment of the necessary cofactors to alter gene expression.

#### Animal models.

Animal models illustrate well the central importance of HIF in regulating the hypoxic behavior of the pulmonary circulation. Mice heterozygous for deletion of HIF1α demonstrate attenuated physiological sequelae of alveolar hypoxia (105), with delayed polycythemia, pulmonary hypertension, and right ventricular hypertrophy. Animals heterozygous for deletion of HIF2α are similarly protected, and do not show the sizeable increases in pulmonary expression of endothelin-1 or the rise in plasma catecholamine concentration, both of which are evident during prolonged hypoxia in wild-type littermates (18). Additionally, mice heterozygous for a gain-of-function mutation that activates HIF2α (initially identified in patients with unexplained erythrocytosis) develop pulmonary hypertension under euoxia in a gene dose-dependent manner (92). In terms of the importance of the HIF pathway for ventilation, mice heterozygous for deficiency of PHD2 (one of three closely-related PHD enzymes) demonstrate enhanced ventilatory sensitivity to acute hypoxia, similar to that seen in wild-type animals exposed to sustained hypoxia for a week in whom acclimatization has occurred. Enlargement of the carotid bodies is also a feature of these animals (16).

#### Chuvash polycythemia: upregulated hypoxia sensing exemplified.

A rare human genetic condition, Chuvash polycythemia (CP), provides a striking demonstration of the importance of the HIF pathway. When either of two specific proline residues in HIFα is hydroxylated, this permits binding of the von Hippel-Lindau protein (VHL), an essential step in the process leading to proteasomal degradation of HIFα (40, 41). Patients with CP are homozygous for a mutation leading to the production of a hypomorphic form of VHL which does not bind hydroxylated HIFα as strongly (5). Thus, at normal oxygen tensions, these individuals have a HIF system signaling hypoxia as a consequence of inappropriate HIFα stabilization. This condition is distinct from the von Hippel-Lindau familial cancer syndrome, an autosomal dominant condition characterized by a variety of malignancies, in which grossly disturbed hypoxia physiology is not a feature (26).

**Fig. 2.**
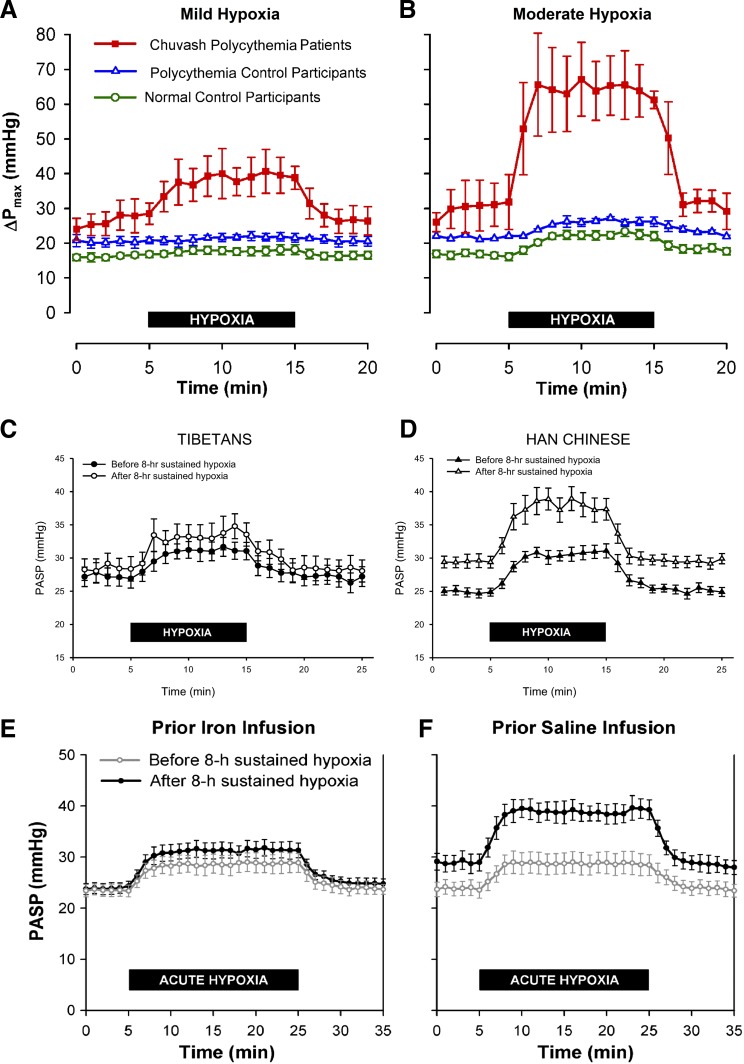
*A* and *B*: pulmonary vascular responses to mild (Pet_O_2__ = 70 mmHg) and moderate (Pet_O_2__ = 50 mmHg) isocapnic hypoxia in patients with Chuvash polycythemia. Control groups comprised healthy volunteers and patients with polycythemia rubra vera. ΔPmax increased by 11.5 mmHg in the Chuvash polycythemia group during mild hypoxia, compared with only 1.1 mmHg in the normal control group (*P* < 0.05). Moderate hypoxia stimulated a rise in ΔPmax of 35.3 vs. 6.1 mmHg, respectively (*P* < 0.001). *C* and *D*: PASP responses to acute isocapnic hypoxia before and after an 8-h exposure to sustained isocapnic hypoxia. Data are for 10 Tibetan and 10 Han Chinese volunteers. *E* and *F*: the effect of 8-h isocapnic hypoxia on the rise in PASP during an acute isocapnic hypoxic challenge, and the attenuating effect of intravenous iron. With a prior saline infusion, following 8-h hypoxia there is an elevated euoxic PASP and an exaggerated response to a 20-min hypoxic challenge. With a prior iron infusion, euoxic PASP is unaffected following 8-h hypoxia, and the response to an acute hypoxic challenge is only slightly augmented. The intravenous iron has all but abolished the acclimatization effect on the pulmonary circulation. All values are means; error bars represent SE. [Modified from Smith et al. (82), Petousi et al. (63), and Smith et al. (81) with permission.]

CP is characterized by polycythemia, pulmonary hypertension, and elevated ventilation at normal alveolar oxygen tensions. Exposure of individuals with CP to even mild degrees of alveolar hypoxia results in a marked increase in PASP (Fig. 2*A*) and ventilation (82). At oxygen tensions producing appreciable effects in healthy volunteers, CP patients demonstrate very profound responses; the rise in PASP is so great as to bring pressures close to those in the systemic circulation (Fig. 2*B*). Patients with CP are, therefore, similar in many ways to healthy individuals who have acclimatized after a prolonged period at altitude (83).

#### Turning-down hypoxia sensing: advantageous at altitude?

No human diseases characterized by a pathological downregulation of the HIF pathway have yet been identified. However, mutations affecting the pathway in this manner may be advantageous for populations resident at high altitude, for example by protecting against the exaggerated polycythemia and pulmonary hypertension seen in chronic mountain sickness (CMS).

Tibetans are one population living at altitude for a period of time sufficiently long for a selection pressure to have had an appreciable effect (64). The Tibetan Plateau has probably been inhabited for at least 25,000 years (2, 66), exposing this group to an inspired partial pressure of oxygen of around 80 mmHg. Very soon after genetic approaches were applied to this population, multiple single-nucleotide polymorphisms (SNPs) related to the gene encoding HIF2α, known as *EPAS1*, were identified as having undergone positive selection (13, 103). HIF2α is one of three HIFα paralogues in humans. Sex-adjusted hemoglobin concentrations at altitude in Tibetan individuals homozygous for the major alleles were almost 1 g/dl lower than heterozygotes (13). A study of Tibetans living in the United Kingdom confirmed it was not just erythropoiesis that was affected; compared with a control group of Han Chinese individuals, Tibetans exhibited hyporeactivity of the pulmonary circulation in response to an acute (10-min) hypoxic challenge, as well as diminished acclimatization of the pulmonary vasculature during a sustained (8-h) hypoxic exposure (Fig. 2, *C* and *D*).

Interestingly, SNPs related to the gene *EGLN1*, which encodes PHD2, have also been identified as having undergone positive natural selection in the Tibetan population (80, 103) and are similarly associated with lower hematocrit at altitude. Presumably the effect of these genetic differences is opposite to that seen in CP patients, such that intracellular HIFα concentrations are lower, either as a result of reduced basal transcription of HIFα mRNA, or more rapid proteasomal degradation of the HIFα protein at a given oxygen tension. Recent work studying Tibetan individuals resident in the United States has provided evidence supporting the latter effect. Missense mutations in exon 1 of *EGLN1* give rise to a PHD2 variant with a lower *K*m for oxygen (52). As a result, this mutant HIF-hydroxylase may promote increased HIFα hydroxylation and degradation compared with the wild-type enzyme, serving to “damp down” hypoxia signaling.

## INFLUENCING OXYGEN SENSING BY MANIPULATING IRON BIOAVAILABILITY

The various genetic effects on canonical HIF pathway signaling described above, as well as others that have been identified (26, 49), must act by altering the relationship between oxygen tension and binding of functional HIFα-HIFβ dimers to HREs. There is considerable interest in pharmacological manipulation of the HIF-hydroxylases (59), particularly in the production of therapeutic inhibitors of PHDs. One application is in the treatment of anemia resulting from chronic renal disease. In patients with fibrotic kidneys who are receiving hemodialysis, endogenous erythropoietin production can be greatly augmented using a small molecule PHD inhibitor, implicating disordered oxygen sensing, rather than simply loss of erythropoietin production capacity, in the etiology of renal anemia (15). Aside from this avenue of investigation, the question arises as to whether there may be biological factors that disturb the relationship between oxygen tension and HIFα protein activity. One such factor of particular interest is iron (56).

### 

#### HIF-hydroxylases and ferrous iron.

The HIF-hydroxylases are members of the 2-oxoglutarate and iron-dependent dioxygenase superfamily (51). The ferrous iron required as a cofactor in these enzymes was recognized as being particularly prone to displacement and oxidation, leading to the suggestion that HIF-hydroxylase activity was likely to be sensitive to intracellular iron availability (74). In vitro, iron chelation with desferrioxamine (DFO) induces HIF1 activity and erythropoietin mRNA expression with a similar time course to hypoxia (97). This iron dependence may explain the long recognized hypoxia-mimetic effect of cobalt ions, including an erythropoietic effect in humans (14). It is possible that cobalt substitutes for the ferrous iron in HIF-hydroxylases, inhibiting them and stabilizing HIFα, although this has not been conclusively shown to occur in vivo, and several other possible mechanisms exist (54). Further in vitro studies have clearly demonstrated the marked sensitivity of PHD activity to intracellular iron availability (47, 61), although it remains difficult to prove directly that this is due to the requirement of these enzymes for ferrous iron as a cofactor, as opposed to an effect on intracellular redox status, for example.

#### Human laboratory studies of iron depletion and supplementation.

In studies of healthy human volunteers, DFO infusion was shown to increase serum erythropoietin under euoxic conditions in a dose-dependent fashion (67) as well as elevating euoxic PASP (10), the latter effect with a very similar time course to HPV. The response of the pulmonary vasculature to an acute hypoxic challenge is also considerably enhanced following a prolonged infusion of DFO (81). The clinical management of patients with CP includes venesection to reduce hematocrit, which provides an opportunity to examine the effects of iron depletion on oxygen sensing. Studies of peripheral blood mononuclear cells from CP patients with low ferritin suggest that iron deficiency does have a significant effect on HIF-target gene expression, albeit in this unusual setting where HIFα is constitutively stabilized (106).

Conversely, increasing iron bioavailability to supraphysiological levels in healthy humans using intravenous iron diminishes HPV (81). In this study, brief hypoxic challenges were employed to measure the responsiveness of the pulmonary circulation before and after a period of acclimatization induced by a long hypoxic exposure. After an 8-h period of eucapnic hypoxia, healthy volunteers demonstrated an increase in euoxic PASP and an exaggerated response to the acute hypoxic challenge compared with before the exposure. As Fig. 2, *E* and *F*, illustrates, when intravenous iron was given in advance of the hypoxic acclimatization period, the usual elevation in euoxic PASP was abolished and the response to the acute hypoxic challenge was only slightly greater than at the outset (81).

**Fig. 3.**
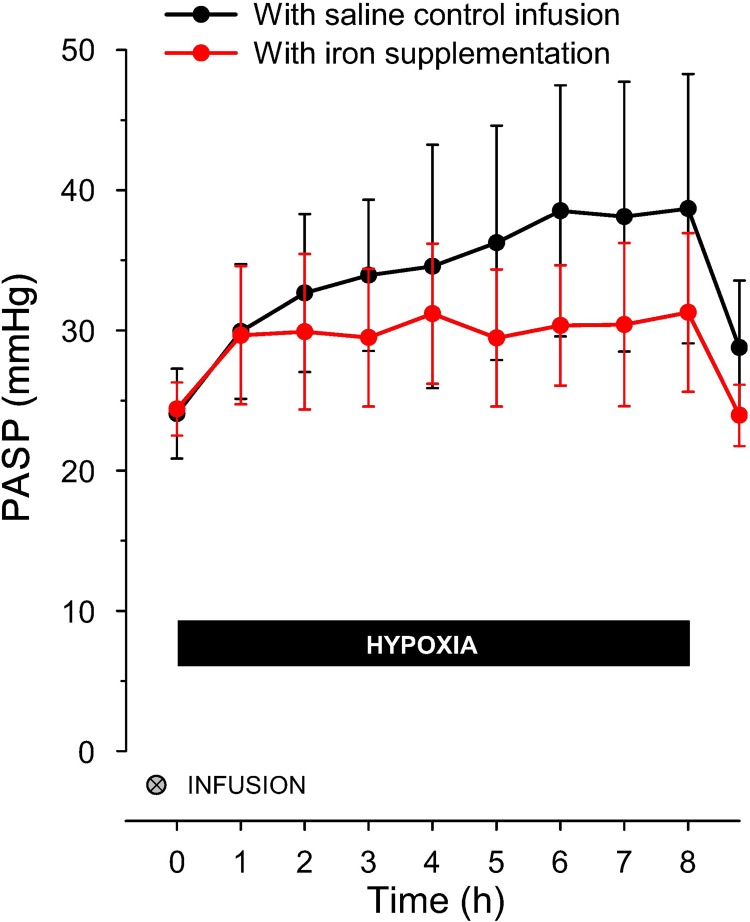
Change in PASP during the 8-h hypoxic exposure in the same individuals as in Fig. 2, *E* and *F*. Acute responses are similar with or without iron loading, but the progressive rise in PASP with continued hypoxia is abolished by iron. Values are means; error bars represent SD. [Modified from Talbot et al. (89) with permission.]

Following intravenous iron, the time course of the progressive rise in PASP during the long hypoxic exposure, as shown in Fig. 3, is also fundamentally different (89). It is as if intravenous iron abolishes the second phase of HPV, along with its consequences for the subsequent behavior of the pulmonary vasculature on return to euoxia, while having very little, if any, effect on the first phase of HPV. In some respects, intravenous iron loading causes the response of the pulmonary vasculature to hypoxia to resemble that observed in Tibetans (27), further supporting the hypothesis that iron is acting through HIF in these human studies.

**Fig. 4.**
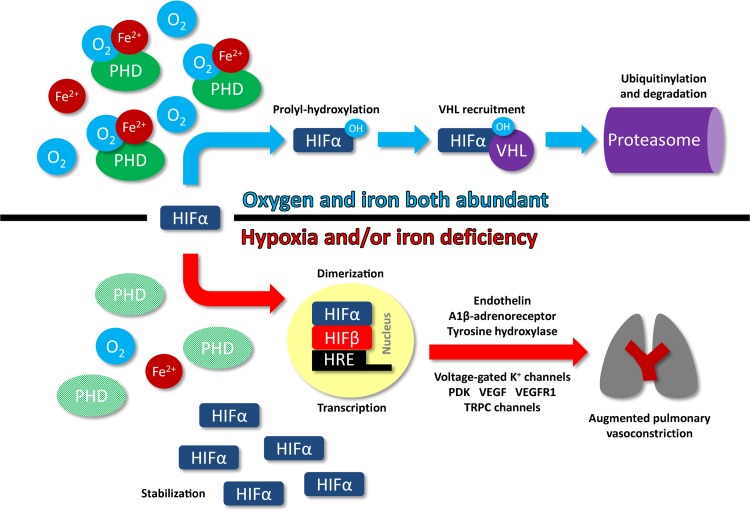
The alternative fates of HIFα according to iron and oxygen availability, and examples of HIF-regulated gene products that influence the pulmonary vasculature (74, 75). HRE, hypoxia-response element; OH, hydroxyl group; PDK, pyruvate dehydrogenase kinase; PHD, prolyl-hydroxylase domain-containing enzyme; VEGF, vascular endothelial growth factor; VEGFR1, VEGF receptor 1; VHL, von Hippel-Lindau protein; TRPC, transient receptor potential classical.

Although there is good reason for believing that the effects of iron described here are mediated via actions on the HIF pathway, the human studies described above are not a direct proof. For example, large doses of intravenous iron may have an effect on HPV via HIF-independent mechanisms, such as a direct effect on potassium channels from free radical generation. A further study in our laboratory suggests, however, that HPV may be attenuated chronically using repeated small doses of intravenous iron to elevate body iron stores, to a similar magnitude as when one large dose is given (Bart NK, unpublished observations). This finding would not be in keeping with an effect mediated by high serum non-transferrin-bound iron levels. Direct evidence that iron deficiency elevates HIF in the lung is provided by the finding that rats fed a profoundly iron-deficient diet show increased lung expression of HIF1α and HIF2α, and resultant upregulation of HIF-target genes (19). Moreover, pulmonary arterial hypertension and right ventricular hypertrophy develop in these animals, and these abnormalities are ameliorated by iron replacement. Candidate human HIF-regulated genes that may contribute to the development of hypoxic pulmonary hypertension include those coding for endothelin, voltage-gated potassium channels, transient receptor potential calcium channels, and the sodium-hydrogen antiporter (75). Figure 4 gives an overview of the putative action of iron deficiency on the pulmonary vasculature, mediated by the HIF pathway.

#### Human studies in the field.

**Fig. 5.**
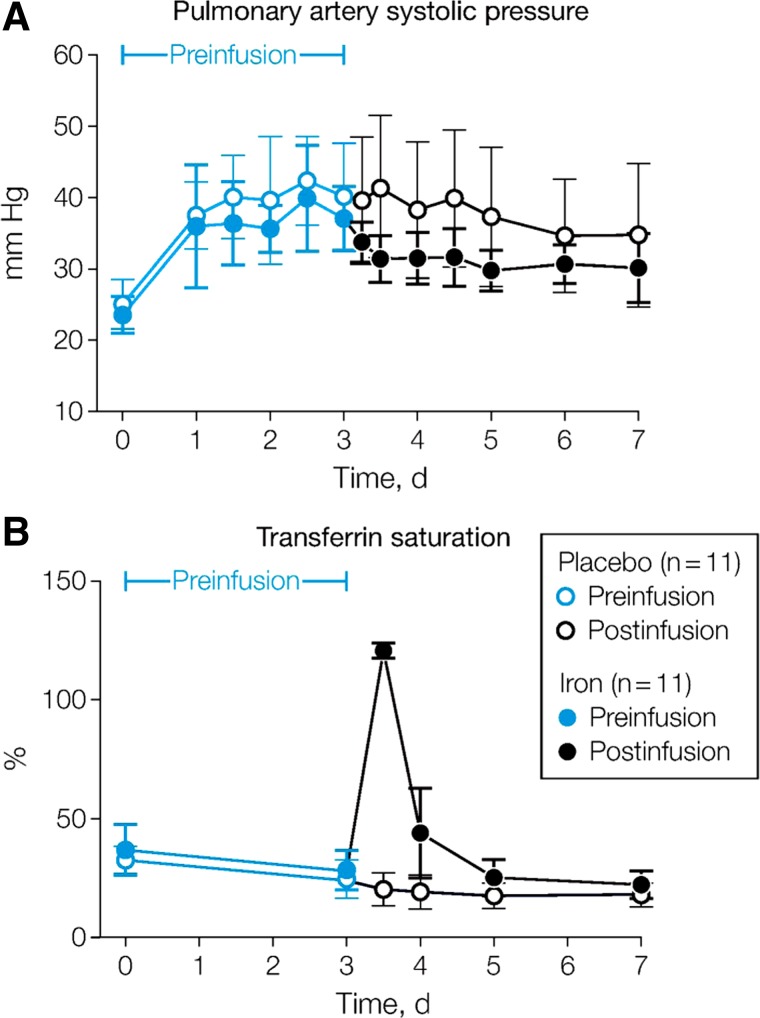
PASP (*A*) and serum transferrin saturation (*B*) over 1 wk following ascent to 4,340 m in Peru in 2 groups of 11 healthy male sea-level residents. Intravenous iron-sucrose (200 mg) or placebo was administered on *day 3*. [Modified from Smith et al. (84) with permission.]

The work described so far was conducted under laboratory conditions and employed eucapnic normobaric hypoxia. Clearly there may be differences at altitude due not only to poikilocapnia but also because hypobaric hypoxia may have subtly different effects from normobaric hypoxia (55). A study in Peru (84) that took healthy individuals from sea level to 4,340 m and measured PASP over a week found, as expected, a significant rise in PASP induced by hypobaric hypoxia shortly after ascent. After 3 days at this elevation, individuals were randomized in a blinded fashion to receive either intravenous iron (200 mg iron-sucrose) or saline. Those given iron showed a significant fall in PASP by 4 h; overall ∼40% of the pulmonary hypertensive response to hypobaric hypoxia was reversed by iron. Importantly, this effect was not simply evident during the phase of grossly elevated circulating iron levels (Fig. 5). This suggests that the effect of intravenous iron on the pulmonary circulation may be sustained for a sufficient length of time to make it useful clinically. More recent data from our laboratory support the view that the effects of a single dose of intravenous iron (ferric carboxymaltose 15 mg/kg; maximum 1 g) on HPV are sustained at a clinically relevant level for a period of weeks to months (Bart NK, unpublished observations).

#### Possible adverse effects of intravenous iron.

One concern is that the doses of iron administered in these human physiology studies are large and may cause adverse effects from, for instance, free-radical generation. It is certainly the case that in patients with chronic renal failure given a single dose of intravenous iron (100 mg iron-sucrose), evidence of oxidative stress and transient proteinuria can be demonstrated (1). However, a study giving repeated doses of intravenous iron (200 mg iron-sucrose weekly for 5 wk) to patients with chronic heart failure, renal impairment, and iron-deficiency anemia, found that iron-loading in fact resulted in a fall in the inflammatory marker C-reactive protein and improved renal function (93). This study, along with others in chronic cardiopulmonary disease that are discussed later, do not support the view that oxidative stress from intravenous iron administration translates into a clinically meaningful deleterious effect, although as newer intravenous iron preparations are increasingly used in clinical practice we can expect to accumulate more evidence in this regard.

## IRON AND HPV IN HIGH-ALTITUDE ILLNESS

There is good reason to suspect that iron may be important in the pathogenesis of high-altitude illness (HAI). Individuals prone to high-altitude pulmonary edema (HAPE) exhibit, as a group, more potent HPV than those who are not HAPE-susceptible (33). One possibility is that differences in iron bioavailability contribute to this interindividual variation. The erythropoietic drive which results from ascent to altitude is a large sink for iron and this may induce iron deficiency as a consequence (69), even in the face of apparently adequate baseline iron stores. In Fig. 5 a fall in transferrin saturation can be observed over the first 3 days at high altitude in both groups, reflecting a change in the balance between iron supply and demand. A recent study has examined the relationship between serum iron, HAPE and the hormone hepcidin, which negatively regulates the availability of circulating iron (4). This study demonstrated an elevation of hepcidin associated with inflammation in HAPE, but failed to show any difference in serum iron between those with HAPE and those without. Whether or not iron status contributes to the pathogenesis of HAPE, a mechanism by which it could contribute to the “unevenness” of HPV—implicated in the pathogenesis of HAPE (12, 37)—is not immediately obvious.

With respect to acute mountain sickness (AMS), in a further study in Peru (91), healthy individuals who were rapidly transported from sea-level to 4,340 m had a smaller increase in Lake Louise score on the first day at high altitude if given iron before ascent. In patients with existing CMS, iron depletion induced by repeated venesection was associated with a gradual rise in PASP, although interestingly this was not immediately reversed by intravenous iron infusion (84).

Although a strong hypoxic pulmonary vasoconstrictor response may predispose to HAI, a strong hypoxic ventilatory drive may confer some protection. While the HIF-system may influence the magnitude of both responses, as is illustrated by patients with CP (82), in none of the human studies discussed so far has an effect of iron status been found on the ventilatory response to hypoxia. It is also of note that no correlation was evident between the magnitudes of these two responses in a study of early acclimatization in 80 individuals (24). There are of course many potential explanations, including different relative contributions of the two main HIFα paralogues and differences in regulation of cellular iron homeostasis between the two systems.

## IRON AND THE PULMONARY CIRCULATION IN CHRONIC DISEASE

Nutritional iron deficiency is extremely common worldwide, affecting perhaps two billion individuals (107). Additionally, in many chronic diseases a state of functional iron deficiency or iron sequestration exists; there are sufficient stores of iron in the body but these are inaccessible for the processes that require it, most obviously erythropoiesis. Inflammation, which elevates circulating levels of hepcidin, is a major etiological factor (28, 99). Given the prevalence of iron deficiency, even a small effect of iron status on oxygen sensing would be potentially significant for human health. Preliminary data from our laboratory suggest that iron deficiency in otherwise healthy individuals does indeed influence HPV (Frise MC, unpublished observations).

### 

#### Chronic heart failure and idiopathic pulmonary arterial hypertension.

Iron deficiency has received considerable attention in the setting of two chronic cardiopulmonary conditions: chronic heart failure (CHF) and pulmonary arterial hypertension. In CHF, iron deficiency is a strong predictor of mortality that is independent of anemia (46). In two large randomized-controlled trials, treating iron-deficient CHF patients with intravenous iron produced very significant improvements in functional outcomes independent of any effect on hemoglobin (6, 65). More recently, iron deficiency has been shown to predict death in patients following an episode of acute heart failure (43), again independently of anemia. Remarkably, any explanation for how iron deficiency confers this excess risk of mortality has been lacking. Iron has pleotropic functions in human biology, but disordered oxygen sensing appears to be one possibility.

In idiopathic pulmonary arterial hypertension (IPAH), iron deficiency is common (85) and correlates with hemodynamic measures (71) as well as WHO functional class (68). Here, mutations that disturb bone morphogenetic protein signaling underlie a significant proportion of cases, and this pathway is instrumental in iron homeostasis in its own right (94). A very small study has recently suggested intravenous iron therapy may be helpful in IPAH (95), and a much larger randomized-controlled clinical trial involving comprehensive clinical assessment of these individuals is also underway (39).

#### Chronic obstructive pulmonary disease.

One condition with a very high global burden of morbidity and mortality, in which iron deficiency has received less attention, is chronic obstructive pulmonary disease (COPD). This disorder is of particular interest because inflammation, chronic hypoxemia, and hypoxia-driven pulmonary hypertension are all present. In view of this, a degree of iron deficiency would be expected, and additionally two small studies have suggested that anemia due to iron deficiency may be common in such patients (73, 79). A small controlled study from our laboratory has recently found that iron deficiency, even in the absence of anemia, is common in patients with COPD, and shows a complex association with inflammation and hypoxemia (60).

## IRON-RESPONSIVE ELEMENTS: A FURTHER LINK BETWEEN IRON AND HIF

This review has focused on the HIF pathway as a potential mediator of the effects of iron upon oxygen-sensing behavior, particularly HPV. Indeed, given the iron dependence of the HIF-hydroxylases, the function of the HIF pathway could equally well be viewed as concerned with sensing and signaling levels of intracellular iron in a manner modulated by oxygen. However, intracellular responses to iron are also regulated through iron-responsive elements (IREs) and the proteins that bind to them (IRPs). Binding of iron to IRPs induces a conformation change that inhibits their interaction with IREs. Unlike HREs, which transcriptionally regulate gene expression, IREs are found in the 5′- and 3′-untranslated regions of various mRNAs and exert translational control over a number of genes important in iron homeostasis. These include ferritin (9, 36), ferroportin (53), and divalent metal transporter 1 (34). Recently it was recognized that HIF2α mRNA contains an IRE in the 5′-region (72), although HIF1α does not. The effect of IRP1 binding to the IRE in HIF2α is inhibitory. The posttranslational control of HIF2α degradation and activity by HIF-hydroxylases, and the translational regulation of HIF2α protein synthesis by IRE-IRP, are two opposing regulatory mechanisms. Deletion of IRP1 in mice leads to splenomegaly and extramedullary hematopoiesis (102) since marked iron deficiency no longer constrains HIF2α translation. When these mice are fed an iron-deficient diet, erythropoiesis is paradoxically stimulated by augmented erythropoietin synthesis. Importantly from the perspective of this review, spontaneous pulmonary hypertension is a prominent feature in these animals (30). It is notable that none of the studies of the HIF or PHD2 knockout animal models discussed early on in this review (16, 18, 92, 105) specifically addressed the consequences for iron homeostasis of these genetic alterations, which may be relevant in their own right.

Further discussion of the complex interactions between the HIF and IRE-IRP systems is beyond the scope of this review. It is worth pointing out, however, that the in vitro and in vivo studies of iron chelation and supplementation collectively presented here do suggest that modulation of HIF-hydroxylase activity is the dominant mechanism through which iron affects HIF-regulated gene expression. For a more detailed discussion of these issues, the interested reader is directed to the accompanying article by Gassmann and Muckenthaler (29).

## CONCLUSION

This mini-review has outlined a number of studies addressing the role of the HIF pathway in modulating HPV, and the manner in which it may be influenced by iron. There is much that is unknown, but the role of iron in this pathway is not only of physiological interest but real clinical significance.

## GRANTS

Work in the authors' laboratory is supported by the National Institute for Health Research (NIHR) Oxford Biomedical Research Centre Programme. The views expressed are those of the authors and not necessarily those of the NHS, the NIHR, or the Department of Health. M. C. Frise is supported by a British Heart Foundation Clinical Research Training Fellowship (FS/14/48/30828).

## DISCLOSURES

P.A.R. has received funding from Vifor Pharma, the manufacturers of Ferinject ®, an intravenous iron preparation, for research studies not presented in this review.

## AUTHOR CONTRIBUTIONS

Author contributions: M.C.F. prepared figures; M.C.F. drafted manuscript; M.C.F. and P.A.R. edited and revised manuscript; M.C.F. and P.A.R. approved final version of manuscript.

## Glossary

AMS: Acute mountain sickness
CHF: Chronic heart failure
CO: Cardiac output
COPD: Chronic obstructive pulmonary disease
CMS: Chronic mountain sickness
CP: Chuvash polycythemia
DFO: Desferrioxamine
EPAS1: Endothelial PAS domain-containing protein 1
EGLN1: Egl nine homolog 1
HAI: High-altitude illness
HAPE: High-altitude pulmonary edema
HIF: Hypoxia-inducible factor
HPV: Hypoxic pulmonary vasoconstriction
HRE: Hypoxia-response element
IPAH: Idiopathic pulmonary arterial hypertension
IRE: Iron-responsive element
IRP: Iron-regulatory protein
PASP: Pulmonary arterial systolic pressure
PDK: Pyruvate dehydrogenase kinase
PHD: Prolyl-hydroxylase domain
PVR: Pulmonary vascular resistance
RAP: Right atrial pressure
TRPC: Transient receptor potential classical
VEGF: Vascular endothelial growth factor
VHL: von Hippel-Lindau
